# An Advanced Human Intestinal Coculture Model Reveals Compartmentalized Host and Pathogen Strategies during *Salmonella* Infection

**DOI:** 10.1128/mBio.03348-19

**Published:** 2020-02-18

**Authors:** Leon N. Schulte, Matthias Schweinlin, Alexander J. Westermann, Harshavardhan Janga, Sara C. Santos, Silke Appenzeller, Heike Walles, Jörg Vogel, Marco Metzger

**Affiliations:** a Institute of Molecular Infection Biology (IMIB), University of Würzburg, Würzburg, Germany; b Institute for Lung Research, Philipps University, Marburg, Germany; c Department of Tissue Engineering and Regenerative Medicine, University Hospital Würzburg, Würzburg, Germany; d Helmholtz Institute for RNA-Based Infection Research (HIRI), Helmholtz Centre for Infection Research (HZI), Würzburg, Germany; e Comprehensive Cancer Center Mainfranken, University of Würzburg, Würzburg, Germany; f Core Facility Tissue Engineering, University of Magdeburg, Magdeburg, Germany; g Fraunhofer Institute for Silicate Research ISC, Translational Centre for Regenerative Therapies TLC-RT, Würzburg, Germany; Department of Veterinary Medicine

**Keywords:** *Salmonella*, gene expression, infectious disease

## Abstract

A major obstacle in infection biology is the limited ability to recapitulate human disease trajectories in traditional cell culture and animal models, which impedes the translation of basic research into clinics. Here, we introduce a three-dimensional (3D) intestinal tissue model to study human enteric infections at a level of detail that is not achieved by conventional two-dimensional monocultures. Our model comprises epithelial and endothelial layers, a primary intestinal collagen scaffold, and immune cells. Upon *Salmonella* infection, the model mimics human gastroenteritis, in that it restricts the pathogen to the epithelial compartment, an advantage over existing mouse models. Application of dual transcriptome sequencing to the *Salmonella*-infected model revealed the communication of epithelial, endothelial, monocytic, and natural killer cells among each other and with the pathogen. Our results suggest that *Salmonella* uses its type III secretion systems to manipulate STAT3-dependent inflammatory responses locally in the epithelium without accompanying alterations in the endothelial compartment. Our approach promises to reveal further human-specific infection strategies employed by *Salmonella* and other pathogens.

## Abstract (precis)

Infection research routinely employs *in vitro* cell cultures or *in vivo* mouse models as surrogates of human hosts. Differences between murine and human immunity and the low level of complexity of traditional cell cultures, however, highlight the demand for alternative models that combine the *in vivo*-like properties of the human system with straightforward experimental perturbation. Here, we introduce a 3D tissue model comprising multiple cell types of the human intestinal barrier, a primary site of pathogen attack. During infection with the foodborne pathogen Salmonella enterica serovar Typhimurium, our model recapitulates human disease aspects, including pathogen restriction to the epithelial compartment, thereby deviating from the systemic infection in mice. Combination of our model with state-of-the-art genetics revealed *Salmonella*-mediated local manipulations of human immune responses, likely contributing to the establishment of the pathogen’s infection niche. We propose the adoption of similar 3D tissue models to infection biology, to advance our understanding of molecular infection strategies employed by bacterial pathogens in their human host.

## INTRODUCTION

*Enterobacteriaceae* are major commensals of the human gut microflora, but certain members of this bacterial family, particularly *Escherichia*, *Salmonella*, *Shigella*, and *Yersinia* species, cause a range of different infections that sum up to millions of cases annually (1). Of these latter pathogens, Salmonella enterica serovar Typhimurium (henceforth *S.* Typhimurium) is a major research model for bacterial virulence strategies and host defense mechanisms during enteric infections. Host cell infection by *S.* Typhimurium depends on the concerted activity of effector proteins encoded on dedicated genomic virulence loci, referred to as *Salmonella* pathogenicity islands (SPIs) (2, 3). The two major SPIs (SPI1 and SPI2) additionally encode structural components of type III secretion systems (T3SSs) that deliver the virulence effector cocktail into the host cytosol. While the SPI1 T3SS and associated effectors mediate epithelial cell invasion (4), intracellular survival is promoted by virulence genes associated with the SPI2 cluster (5). Host cell manipulations mediated by SPI1 and SPI2 effectors include rearrangements of the actin cytoskeleton, manipulation of phagosomal maturation, and subversion of host immunity pathways (3).

The immune response to *S.* Typhimurium has been investigated extensively. The innate immune system relies on a variety of pattern recognition receptors (PRRs), which sense conserved pathogen-associated molecular patterns (PAMPs), such as bacterial cell wall components or flagellin, to elicit proinflammatory transcriptional responses. In the intestine, Toll-like receptor 5 (TLR5) recognizes *Salmonella* flagellin, which activates cytokine and chemokine production for the recruitment and activation of professional immune cells, such as NK cells, T cells, and monocytes (6). These cells, in turn, respond by producing, e.g., gamma interferon (IFN-γ) and interleukin-6 (IL-6), which activate the Janus kinase/signal transducer and activator of transcription (JAK/STAT) pathway on a variety of target cells to promote antimicrobial defense and changes to the cellular survival and metabolic programs (7, 8). Other major cytokines produced by the activated epithelium and professional immune cells include IL-1, which promotes NF-κB-dependent immune gene expression (9), and IL-8, which functions as a major chemoattractant for bacterium-engulfing neutrophils (10). Recently, long noncoding RNAs (lncRNAs) were also implicated in the host response to *Salmonella* infection (11, 12). NeST lncRNA, for instance, protects from *Salmonella*-induced lethality in mice by promoting IFN-γ expression (13). *S.* Typhimurium, in turn, may partially evade this host defense through its facultative intracellular lifestyle and adaptation to—and even exploitation of—the inflammatory milieu (14, 15).

Responses to *Salmonella* infections of the human gut necessarily require a fine-tuned interplay between the gut mucosa, the vascular endothelium, and the cells of the gut-associated immune system (16). These complex interactions have remained difficult to mimic in a human cell culture setting. The current understanding of host subversion by *S.* Typhimurium and the countermeasures taken by the immune system was largely deduced from studies with immortalized cell lines or mouse models, i.e., infection models with inherent strengths and weaknesses. Cell line monocultures have proven to be invaluable tools to reveal discrete molecular and cellular mechanisms, but they inevitably neglect the complex division of labor and three-dimensional (3D) fine structure within the inflamed tissue. In addition, critical cell components, such as peripheral immune cells, which affect the infection process, are often missing. Likewise, mice have been an important model to study *Salmonella* infections on a whole-system level, but there are profound differences in antimicrobial immunity and *Salmonella* pathogenesis between rodents and humans (17–19). For example, while *S.* Typhimurium induces self-limiting gastroenteritis in immunocompetent humans, it causes systemic infections and even sepsis in mice (6). Therefore, to better understand the molecular mechanisms underlying intestinal *Salmonella* infection in a human setting, more tailored models are needed.

Sophisticated human three-dimensional (3D) *in vitro* models have recently garnered much attention of scientists in academia, product developers in industry, regulatory authorities, and society in general (20, 21). Examples are primary organoid cultures, rotating-wall vessel approaches, and Transwell-like coculture settings (21). Such models can deliver important information about drug toxicity and the mode of action, pathophysiology, normal biological tissue function, or immune responses (22, 23), prior to *in vivo* and clinical extrapolation. Often, however, the available models omit important cellular components, use artificial cellular growth matrices, or are difficult to standardize. To address the particular problem of artificial matrices to support cell growth, tissue models based on recellularized collagen scaffolds, comprising multiple cocultured cell types to more accurately mimic the epithelial barriers, were developed (20). So far, however, such models have neglected the vascular immune cell component and have not been widely adopted in infection research.

Here, we present an advanced human intestinal barrier model based upon a recellularized porcine collagen scaffold that was infected with the bacterial model pathogen *S.* Typhimurium. This coculture model, encompassing both the endothelial and the epithelial intestinal barriers as well as a natural collagen matrix and professional immune cells, allowed us to study reciprocal host and pathogen cell adaptations during acute *S.* Typhimurium infection. We show that in this model system, in contrast to small-animal models, *S.* Typhimurium infection is restricted to the epithelial layer and does not spread into the vascular compartment, thereby mimicking human disease. Dual transcriptome sequencing (dual RNA-seq), which comprehensively profiles host and pathogen gene expression during bacterial infections, has been successfully applied to infected cell line-based, two-dimensional (2D) monocultures (reviewed in reference 24) and mouse models of infection (25–27). For the first time, we here applied dual RNA-seq to a 3D tissue model to chart mRNA and noncoding RNA expression changes in the communicating, purified host cell types (intestinal epithelial cells [IECs], endothelial cells, monocytes, NK cells) and in *Salmonella*. Our data sets determined STAT3 signaling to be a central host pathway targeted by the pathogen. Using CRISPR/Cas9-edited IECs and *Salmonella* virulence mutants, we show that the T3SS-dependent manipulation of STAT3 locally changes the inflammatory milieu to the benefit of the pathogen but leaves the basolateral milieu unaltered. Thus, 3D infection models may reveal compartmentalized pathogen strategies not visible in conventional human cell cultures. Our dual RNA-seq data may serve the community as an important resource for prioritizing *Salmonella* virulence factors for further investigation and for defining cell type-specific expression signatures of pathogenic attack at the intestinal barrier.

## RESULTS

### An engineered human intestinal tissue model to study *Salmonella* infection.

**FIG 1:**
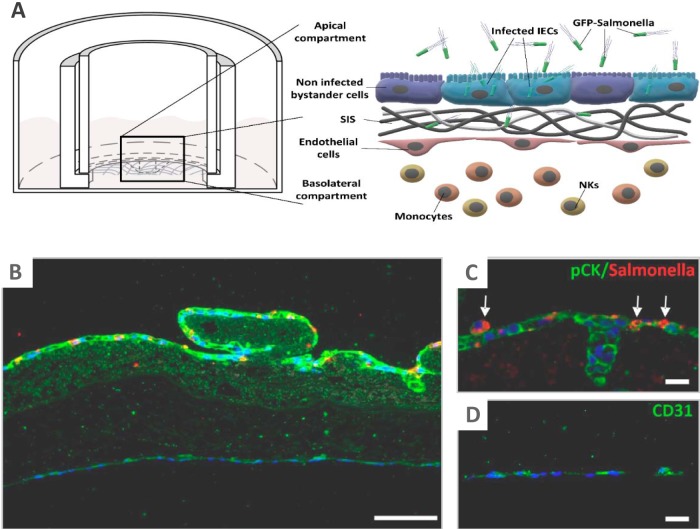
Construction of the intestinal tissue model and experimental layout. (A) (Left) Illustration of a cross-section through a cell crown device within a culture dish, with the collagen membrane being fixed between outer and inner metal rings to create the apical and basolateral compartments. (Right) Schematic representation of the engineered intestinal barrier and experimental setup. The epithelium and endothelium are separated by a collagen layer (SIS, small intestinal submucosa), and leukocytes are supplied into the basolateral compartment. Infection is triggered by addition of GFP-positive *Salmonella* into the apical compartment. (B) Fluorescence microscopy analysis of a cross section through a *Salmonella*-infected intestinal barrier model (24 h postinfection; MOI, 10). The epithelium and endothelium are visualized by pCK and CD31 staining (green), respectively. Nuclei are stained with DAPI (blue). *Salmonella* are stained with an anti-LPS antibody and are shown in red. (C) Magnification showing the epithelial layer with *Salmonella*-infected cells (arrows). (D) Magnification of the endothelial cell layer. Bars, 100 μm (B) or 20 μm (C, D).

Due to the existing limitations in the currently available 3D *in vitro* culture models, infection studies with human-pathogenic bacteria typically neglect the tissue microstructure at the primary site of infection. With regard to the intestinal barrier, its major constituents are the epithelial lining and the underlying collagen scaffold of the lamina propria, harboring blood vessels for nutrient exchange and immune cell recruitment (28). To model the intestinal barrier in a commonly used Transwell-like setting, we fixed a decellularized porcine small intestinal submucosa (SIS) collagen scaffold into a cell crown to obtain two separated compartments (Fig. 1A). The apical compartment was populated with a human intestinal epithelial cell (IEC) line (Caco-2) and matured into a tight epithelial lining. The basolateral surface of the matrix was populated with primary human microvascular endothelial cells, and the underlying separated culture compartment was supplemented with peripheral blood leukocytes as a proxy for the vascular immune system (Fig. 1A).

**FIG 2:**
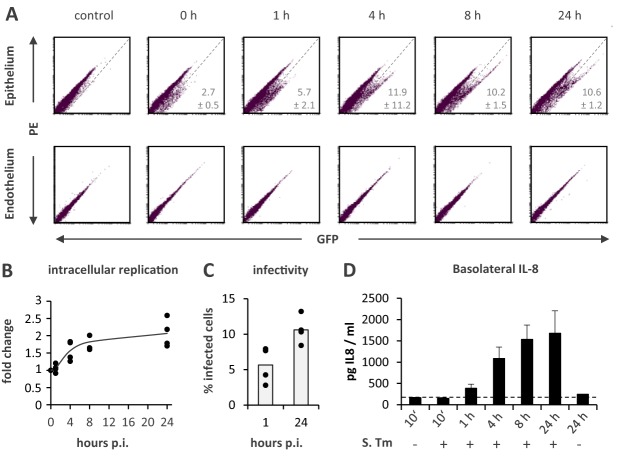
Temporal analysis of *S.* Typhimurium infection of the tissue model. (A) Representative FACS scatterplots showing mock- and *S.* Typhimurium-infected (MOI, 10) epithelial (top row) and endothelial (bottom row) cells in the red (cellular autofluorescence) and green (GFP-expressing *Salmonella*) channels over the course of 24 h. (B) Quantification of the fold increase in the geometric mean green fluorescence intensity comparing the *S.* Typhimurium-infected samples postinfection (p.i.) to the control at 0 h. (C) Quantification of the percentage of infected (GFP-positive) cells at 1 and 24 h postinfection. (D) Quantification of IL-8 cytokine levels in the basolateral compartment upon mock control or *S.* Typhimurium (S. Tm) treatment at the indicated time points via ELISA.

To confirm the suitability of our model for infection studies, we conducted a pilot experiment with a *S*. Typhimurium strain constitutively expressing the green fluorescent protein (GFP) (29) and tracked the bacteria within the tissue construct. Fluorescence microscopy analysis of cross sections visualized epithelial and endothelial cell monolayers, separated by the collagen scaffold, as well as *Salmonella*-infected cells within the epithelium, but not the endothelium (Fig. 1B to D). Flow cytometry of infected models identified a *Salmonella*-positive subpopulation of epithelial but not endothelial cells (Fig. 2A; see also Fig. S1A in the supplemental material), and assays counting the numbers of colony forming units (CFUs) revealed the sterility of the basolateral culture medium (Fig. S1B). In line with these results, no indication for infection of basolateral leukocytes was obtained (Fig. S1C). These results confirm *Salmonella* to be unable to cross the epithelium. The fluorescence signal intensity emitted by invaded epithelial cells increased over time, indicative of *Salmonella* intracellular replication (Fig. 2B) at a rate comparable to previous findings from a 2D Caco-2 infection model (30). Furthermore, an increase in the percentage of invaded cells over time indicated spreading of the infection within the epithelium (Fig. 2C).

10.1128/mBio.03348-19.1 FIG S1 Transmission of *Salmonella* across the collagen barrier. (A) Assays of the numbers of CFU in IEC and endothelial cell lysates from infected tissue constructs at 24 h after the onset of infection relative to the numbers in the bacterial infection inoculum (input). (B) Same as panel A but with apical and basolateral medium at 2 h after the onset of infection (prior to addition of gentamicin). Mean values and standard deviations from independent experiments are shown. (C) Representative FACS plots showing forward and side scatter (FSC and SSC, respectively) plots, CD14 (monocyte) and CD56 (NK-cell) gating, and DAPI/GFP projections of the gated populations for leukocytes recovered from the basolateral compartment of mock-treated or *Salmonella*-infected models at 24 h. Top row, unstained leukocytes from the mock-infected control. Download FIG S1, PDF file, 0.1 MB. Copyright © 2020 Schulte et al. 2020 Schulte et al. This content is distributed under the terms of the Creative Commons Attribution 4.0 International license.

Despite the absence of bacterial transmission across the epithelial barrier, the endothelial cell compartment responded to the infection by release of the major phagocyte attractant IL-8 (Fig. 2D). Thus, our tissue model successfully recapitulates an epithelially retained *Salmonella* infection and immune signaling across the intestinal barrier and thereby resembles human disease, which usually involves gastroenteritis, but no systemic infection, as is observed in mice.

### Processing of *Salmonella*-infected intestinal tissue models for transcriptomics.

**FIG 3:**
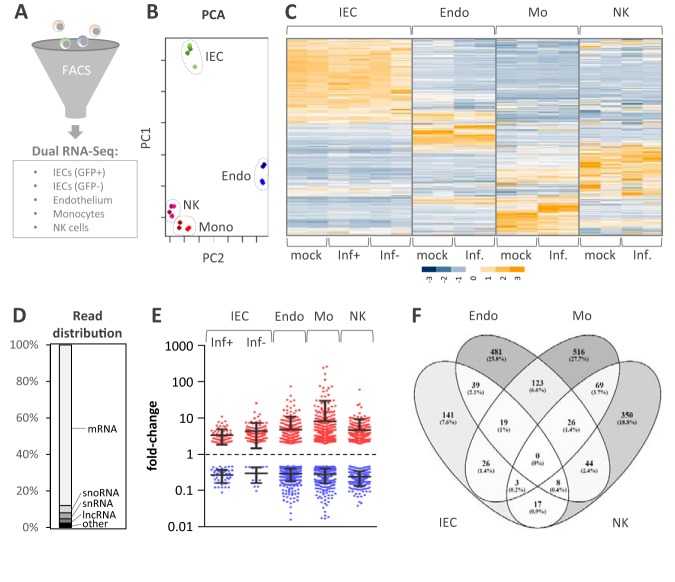
Overview of RNA-seq of the samples and comparison of the global host responses. (A) Experimental scheme. The indicated cell types were purified from the tissue model by FACS and separately analyzed by RNA-seq. IECs were separated into GFP-positive (GFP^+^; *Salmonella*-infected) and GFP-negative (GFP^−^; noninfected) populations and subjected to dual RNA-seq analysis. (B) PCA of RNA-seq libraries, based on row Z-scores. (C) Heat map representation of hierarchical clustering result using RNA-seq data tables from all cell types (row Z-scores, color coded according to the key provided at the bottom). Inf, data obtained from *Salmonella*-infected models; Inf^+^ and Inf^−^, GFP-positive and -negative epithelial cells, respectively, from *Salmonella*-challenged models. (D) Averaged distribution of RNA-seq reads from all libraries over the main RNA classes. (E) Dot-plot representation of gene expression changes (≥2-fold up or down compared to the level of expression by the mock-treated controls) averaged across both replicates and for the indicated conditions. (F) Venn diagram depicting the overlap of regulated genes for which the results are shown in panel E between the different cell types. IEC, intestinal epithelial cells; Endo, microvascular endothelial cells; Mo or Mono, monocytes; NK, natural killer cells.

We sought to utilize our new model to gain an improved understanding of the reprogramming of host immunity by *S.* Typhimurium during infection. To this end, infections were carried out for 24 h with GFP-positive *Salmonella* applied to the apical compartment followed by fluorescence-activated cell sorting (FACS)-based separation of *Salmonella*-invaded epithelial cells (GFP positive) and noninvaded bystander epithelial cells (GFP negative), RNA extraction, rRNA depletion, and dual RNA-seq (Fig. 3A). To follow the propagation of the immune response across the intestinal barrier, cells of the endothelial lining (CD31^+^), monocytes (CD14^+^), and NK cells (CD56^+^) were FACS purified from the same models and their transcriptomes were sequenced. The corresponding cell types from uninfected models served as host controls and the bacterial inoculum served as the *Salmonella* preinfection reference.

Principal-component analysis (PCA) of host cell RNA-seq data (row Z-scores) (Fig. 3B) revealed that samples primarily clustered according to cell type rather than treatment (infected versus noninfected). In line with this observation, specific expression signatures were revealed for IECs, endothelial cells, monocytes, and NK cells (Fig. 3C). Inspection of the detected host transcript classes (Fig. 3D) proved the intended depletion of rRNAs across all cell types. The mRNA fraction occupied ∼88% of all mapped reads, followed by small nucleolar RNAs (snoRNAs; 3.9%), small nuclear RNAs (snRNAs; 3.3%), and lncRNAs (2.3%). In the following, we focus on regulated mRNAs and lncRNAs on the host side. Generally, the host response to *Salmonella* infection (both the number of regulated transcripts and their median fold change in expression) was higher in cells of the basolateral compartment (endothelial cells, monocytes, NK cells) than in cells of the infected epithelium (Fig. 3E and F). This confirms the sensing of the apically retained infection by the vascular components of the tissue construct, as seen in Fig. 2D. Interestingly, the overlap among the infection-regulated host genes between the different cell types was small (Fig. 3F), probably reflecting the nonredundant functions of IECs, endothelial cells, monocytes, and NK cells during bacterial infection.

### Cell type-specific signatures of the vascular immune response.

**FIG 4:**
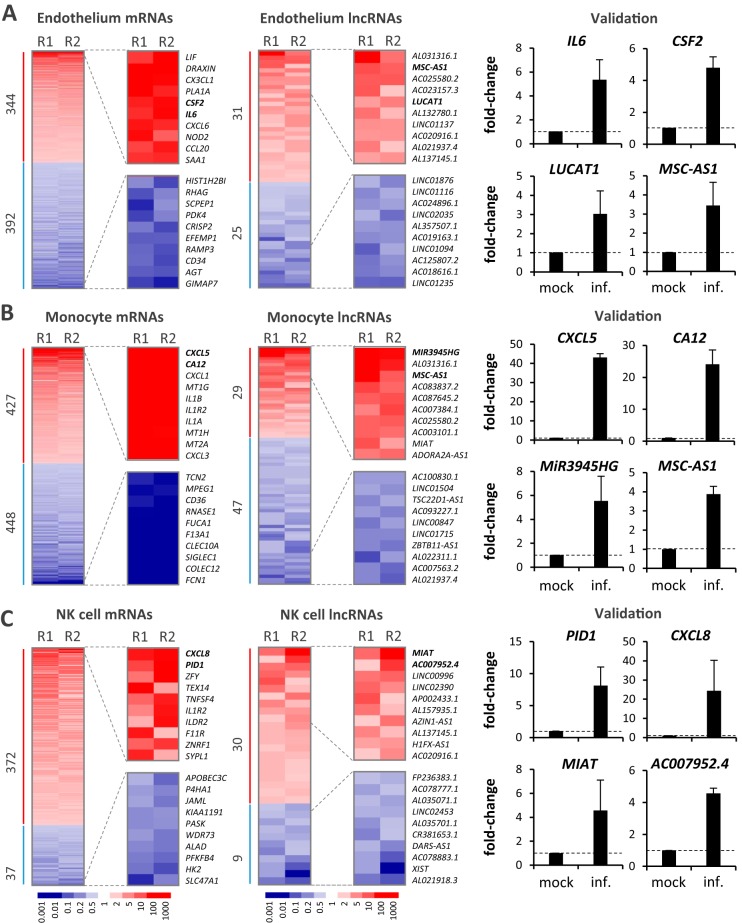
Host gene expression changes in the vascular compartment. (A) Heat map (two experimental replicates, R1 and R2) showing changes in mRNA (left) and lncRNA (middle) expression in endothelial cells upon apical *Salmonella* infection (24 h; MOI, 10) from that obtained by mock treatment of the intestinal tissue construct. The results for the top 10 up- and downregulated genes are shown in magnification to the right of each heat map, and the genes are labeled by name. Fold changes in expression are color coded according to the key provided below panel C. (Right) Validation of the induced expression changes of selected mRNAs and lncRNAs upon infection by qRT-PCR. Combined results (mean ± SD) from three independent experiments are shown. (B) Same as panel A but for monocyte expression data. (C) Same as panel A but for NK-cell data.

To characterize the nature of the respective responses by the four interacting host cell types, we closely inspected mRNA and lncRNA expression changes after infection. First, we sought to characterize the propagation of the response of our tissue model to infection across the intestinal barrier. Despite the absence of *Salmonella* transmission into the vascular compartment, endothelial cells upregulated (fold change [FC] ≥ 2; false discovery rate [FDR] < 0.05) 344 mRNAs and downregulated (FC ≤ 0.5; FDR < 0.05) 392 mRNAs upon apical infection (Fig. 4A). In monocytes, which, in conjunction with lymphocytes, such as NK or T cells, function to orchestrate the peripheral inflammatory response, 427 mRNAs were upregulated and 448 mRNAs were downregulated compared to their regulation in the mock-infected controls (Fig. 4B). In NK cells, which, besides their cytotoxic properties, provide antimicrobial cytokine signals to trigger antibacterial responses and antigen presentation, 372 mRNAs were upregulated and 37 were downregulated upon infection (Fig. 4C).

During an acute response to infection, the endothelium functions to transmit the local immune activation signals into the bloodstream to trigger a systemic response (31). In line with this, among the 10 most highly induced mRNAs were those encoding proinflammatory cytokines and immune cell-recruiting chemokines, such as IL-6, CXCL6, and CXCL3L1 (Fig. 4A). Similarly, monocytes upregulated mRNAs encoding major proinflammatory chemokines and systemically acting cytokines, such as CXCL5, CXCL3, IL-1α, and IL-1β (Fig. 4B). Among the top induced mRNAs in NK cells were those encoding neutrophil attractant IL-8 (CXCL8), endothelial cell attachment protein TNFSF4, or the IL-1α/IL-1β decoy protein IL-1 receptor 2 (IL-1R2) (Fig. 4C), suggesting a vital involvement of NK cells in tuning the vascular innate immune response. The induction of IL-8 was identified to be the common denominator of endothelial, monocytic, and NK-cell responses (Fig. S2). Overall, only a few RNAs were induced in more than one cell type (Fig. S2), illustrating the extensive division of labor during innate immune responses to *Salmonella*.

10.1128/mBio.03348-19.2 FIG S2 Division of labor between endothelial, monocytic, and NK cells. A Cytoskape network plot showing the top 50 induced RNAs (mRNAs and lncRNAs, RNA-seq data) upon *Salmonella* infection for each cell type and the overlap in gene regulation between the three cell types. Download FIG S2, PDF file, 0.2 MB. Copyright © 2020 Schulte et al. 2020 Schulte et al. This content is distributed under the terms of the Creative Commons Attribution 4.0 International license.

Noteworthy was the finding that the response to infection by all three basolateral cell types included the differential expression of dozens of lncRNAs which make up a class of transcripts with emerging functions in vertebrate immunity (32, 33). Regulation of selected lncRNAs in all cell types could be confirmed by quantitative real-time PCR (qRT-PCR) analysis (Fig. 4A to C). These measurements also confirmed MSC-AS1 to be a shared lncRNA marker of immune activation in endothelial cells and monocytes. Together, these results demonstrate extensive rewiring of the coding and noncoding transcriptomes of key human cell types involved in vascular immune activation during intestinal *Salmonella* infection.

### Host-pathogen transcriptomics of the infected epithelium.

**FIG 5:**
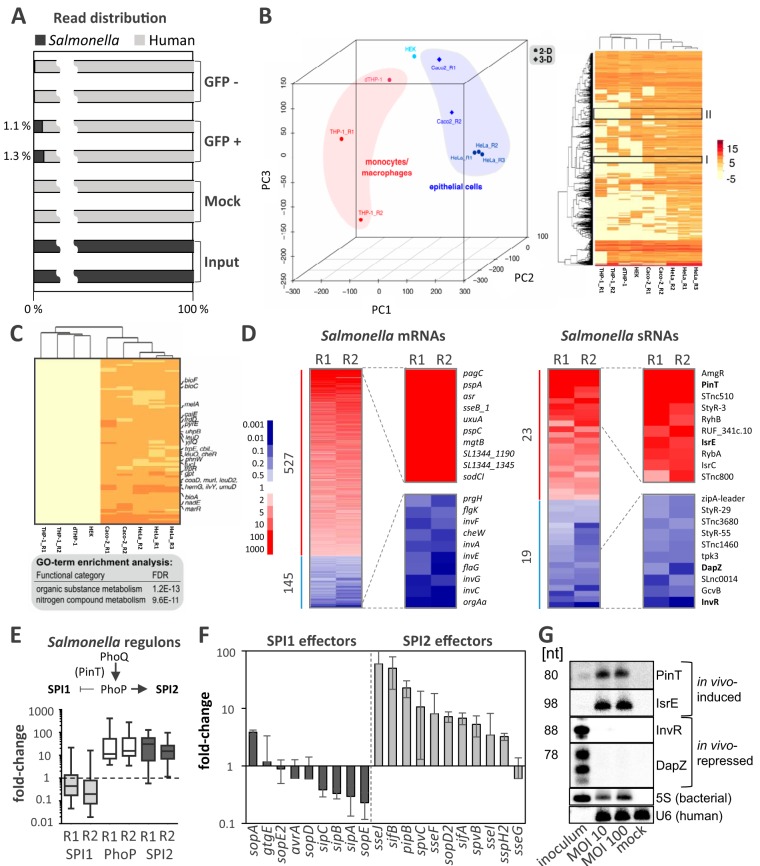
Gene expression of intraepithelial *Salmonella*. (A) Proportion of bacterial reads in the bacterial input sample, mock-treated intestinal epithelial cells (IECs), bystander IECs (GFP negative), and invaded IECs (GFP positive). (B) (Left) PCA of *Salmonella* intracellular transcriptomes from infected THP1 monocytes and macrophages (dTHP1) or the indicated epithelial cell types. Caco-2 cell replicates R1 and R2 are from this study; the rest are from a previous study (30). (Right) Z-score heat map showing *Salmonella* gene expression clusters in the PCA data (for the indicated cell types). Gene clusters (I and II) discriminating epithelial from monocyte/macrophage intracellular *Salmonella* transcriptomes are highlighted. (C) Magnification of cluster I from panel B (right) with enriched GO terms. (D) Heat map (two experimental replicates, R1 and R2) showing changes in mRNA expression (left) and sRNA expression (right) in *Salmonella* inside infected epithelial cells (24 h) from the expression for the input bacterial control. The results for the top 10 up- or downregulated genes are shown in magnification to the right of each heat map, and the genes are labeled by name. Fold changes in expression are color coded according to the key provided on the left. (E) (Top) Schematic illustration of the switch from SPI1 (invasion) to SPI2 (intracellular survival) gene expression upon intracellular activation of the PhoP/Q two-component system. (Bottom) Box plot depicting the regulation of genes belonging to the SPI1, PhoP, and SPI2 regulons in intracellular *Salmonella* compared to the *Salmonella* inoculum. (F) Fold changes in the levels of mRNAs encoding *Salmonella* effectors secreted through the SPI2 or SPI1 T3SS (the mean ± SD was calculated from both RNA-seq replicates). (G) Northern blot validation of the differential expression of *Salmonella* sRNAs prior to infection (inoculum) or 24 h after infection (MOI, 10 or 100). Radioactive signals were absent from mock-infected control samples (mock), supporting the specificity of the selected DNA probes for the intended *Salmonella* transcripts and excluding cross-reactivity with human RNAs. nt, number of nucleotides.

Dual RNA-seq simultaneously records the gene expression of a bacterium and its mammalian host, which allowed us to study reciprocal host-pathogen adaptations during the epithelially retained *S.* Typhimurium infection within FACS-separated IECs. Mapping of RNA-seq reads from the bacterial input and epithelial mock-infected control libraries confirmed almost exclusive alignment to the bacterial or human reference genome, respectively (Fig. 5A). With regard to the infected samples, in the invaded (GFP-positive) but not in the bystander (GFP-negative) epithelial cells, ∼1% of the total reads mapped to the *Salmonella* genome (Fig. 5A), verifying successful separation of infected from noninfected host cells at the cell sorting step. Comparison of these intracellular *Salmonella* transcriptomes to previously recorded expression data for intracellular *Salmonella* within human 2D monocultures (30) by PCA revealed a segregation according to monocytic/macrophage and epithelial cell lineages (Fig. 5B). *Salmonella* genes preferentially expressed during epithelial cell (but not monocyte) infections were enriched for Gene Ontology (GO) terms relating to nitrogen compound metabolism (Fig. 5C). Thus, our intestinal human tissue infection model recapitulates an epithelial cell-adapted *Salmonella* gene expression program, arguing that the intraepithelial environment drives *Salmonella* gene expression largely independently of the presence or absence of additional host cell types in the culture.

Comparison of reads from intraepithelial *Salmonella* to those from the bacterial input sample revealed the upregulation of 527 *Salmonella* mRNAs and the downregulation of 145 *Salmonella* mRNAs (Fig. 5D). Host cell invasion by *Salmonella* requires the activation of genes encoded by the SPI1 locus (4), whereas intracellular survival depends on the expression of genes encoded by SPI2 (5). The switch from SPI1 to SPI2 gene expression involves the PhoP/Q two-component system (34), activation of which is therefore necessary for intracellular survival (35). Accordingly, intracellular *Salmonella* upregulated the expression of genes belonging to the PhoP regulon and SPI2-encoded genes and downregulated the expression of SPI1 genes compared to the gene expression of the bacterial input (Fig. 5E). Host cell manipulation by *Salmonella* occurs through effector proteins secreted through the T3SS encoded on SPI1 and SPI2. In line with the activation of SPI2, expression of SPI2 T3SS-associated effectors, whether encoded on SPI2 itself or within the core genome (except for SseG), was upregulated in *Salmonella* inside flow-sorted IECs (Fig. 5F). On the other hand, SPI1-associated effectors were largely downregulated (Fig. 5F).

Dual RNA-seq also captures the expression of bacterial noncoding transcripts, particularly the class of small noncoding RNAs (sRNAs). Previously, we have uncovered sRNA expression patterns during the intracellular phase of the *Salmonella* infection cycle (30). Confirming that the bacterial expression patterns detected in FACS-enriched GFP-positive IECs indeed reflect intracellular *Salmonella* transcriptome signatures, two PhoP-activated sRNAs, PinT and AmgR (30, 36), were highly induced compared to their expression in the bacterial inoculum (Fig. 5D). Additionally, our data reveal the regulation of dozens of further sRNAs in intracellular *Salmonella* (Fig. 5D; Table S1). For instance, the homologous sRNAs RyhB and IsrE, which are activated under conditions of iron scarcity (37, 38), were strongly induced by intracellular *Salmonella*, in accordance with our previous findings (30). Conversely, among the downregulated sRNAs were members of the SPI1 regulon, such as InvR (39) and DapZ (40). By Northern blotting, we could validate the induction or repression of some of the most strongly regulated sRNAs, as predicted from the dual RNA-seq data (Fig. 5G). Together, these results are in line with the *Salmonella* virulence gene expression patterns previously observed in 2D monoculture models and reflect the adaptation of *Salmonella* to the hostile intracellular milieu within infected IECs.

10.1128/mBio.03348-19.5 TABLE S1 *Salmonella* sRNAs regulated in invaded IECs differently from their regulation in the inoculum. Download Table S1, PDF file, 0.1 MB. Copyright © 2020 Schulte et al. 2020 Schulte et al. This content is distributed under the terms of the Creative Commons Attribution 4.0 International license.

**FIG 6:**
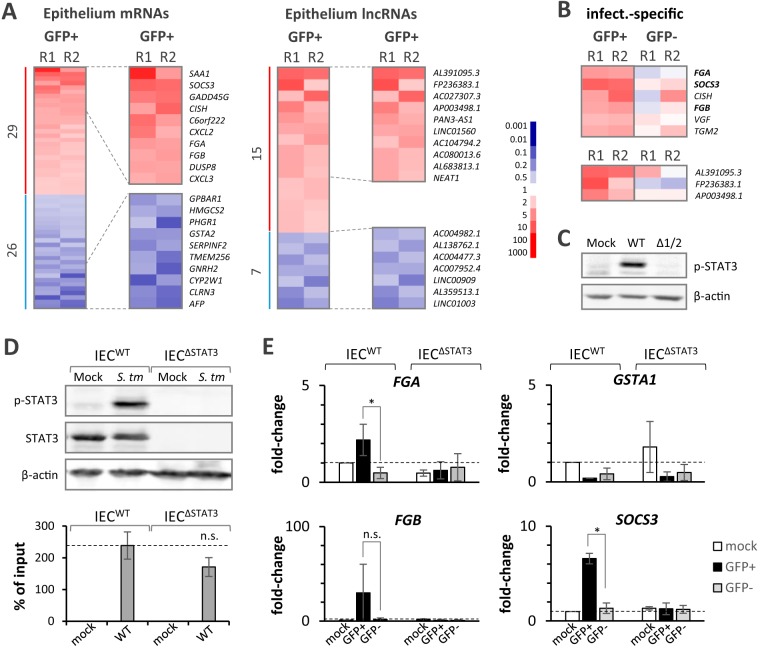
*Salmonella* actively interferes with the epithelial immune response. (A) Heat map analysis (two experimental replicates, R1 and R2) of the changes in mRNA (left) and lncRNA (right) expression in intestinal epithelial cells at 24 h after apical *Salmonella* infection from their expression after mock treatment. The results for the top 10 up- and downregulated genes are shown in magnification to the right of each heat map, and the genes are labeled by name. Fold changes are color coded according to the key provided on the right. (B) Heat map analysis of mRNAs (top) and lncRNAs (bottom) expressed at levels of ≥2-fold higher in *Salmonella*-invaded (GFP-positive) than in bystander (GFP-negative) epithelial cells. Fold changes are color coded according to the key provided in panel A. (C) Western blot analysis of STAT3 phosphorylation induced by wild-type (WT) but not SPI1/2-deficient *Salmonella* (Δ1/2) in intestinal epithelial cells. β-Actin served as a loading control. (D) (Top) Western blot analysis of total and phospho-STAT3 (p-STAT3) in wild-type and STAT3-deficient (ΔSTAT3) intestinal epithelial cells challenged with *Salmonella* or mock treated. β-Actin served as a loading control. (Bottom) Quantification of the *Salmonella* CFU as a percentage of the inoculum recovered from wild-type or STAT3-deficient Caco-2 cells at 24 h postinfection. (E) qRT-PCR validation of *FGA*, *FGB*, *SOCS3*, and *GSTA1* mRNA regulation in *Salmonella*-invaded (GFP-positive) or bystander (GFP-negative) epithelial cells compared to that in the mock-treated controls. Combined results from three independent experiments are shown. *P* values were determined by two-tailed Student's *t* test. *, *P* ≤ 0.05; n.s., not significant.

Since our dual RNA-seq data reflect common patterns of *Salmonella* virulence gene activation preceding the intracellular manipulation of host cell target pathways, we next looked for signs of pathogen-induced alterations in infected IECs. Confirming the initiation of an epithelial immune response by the intestinal barrier model, *Salmonella* infection induced the expression of both coding (*SOCS3*, *CXCL2*, *CXCL3*) and noncoding (*NEAT1* [41]) immune-associated RNAs in IECs (Fig. 6A). Comparison of *Salmonella*-invaded (GFP-positive) to bystander (GFP-negative) epithelial cells revealed genes commonly induced in both cell populations to associate with the tumor necrosis factor and NOD-like receptor innate immune-signaling pathways (Fig. S3B). Bystander cells primarily activated NF-κB- and interferon regulatory factor (IRF)-dependent genes (Fig. S3 and S4), among which IL-17-, chemokine-, and cytokine-signaling pathways were overrepresented. Different from bystander cells, *Salmonella*-invaded IECs showed a specific activation of JAK/STAT3-dependent genes (Fig. S3), including *SOCS3*, *FGA*, and *FGB*, in invaded IECs (Fig. 6B). While suppressor of cytokine signaling 3 (SOCS3) is a major posttranscriptional regulator of immune signaling, fibrinogen alpha and beta (FGA and FGB) are involved in wound healing and direct antimicrobial defense (42) and have recently been revealed to be *Salmonella*-induced STAT3-dependent genes (43). Consequently, Western blot analysis confirmed the previously reported activation of STAT3 phosphorylation by *Salmonella* (30, 43, 44) (Fig. 6C). In line with the known role of secreted *Salmonella* effectors in STAT3 phosphorylation (43), no phosphorylation signal was detected when infections were carried out with a *Salmonella* mutant devoid of the SPI1 and SPI2 pathogenicity islands (strain ΔSPI1/2; Fig. 6C). Together, this confirms that STAT3 signaling is a central host target reprogrammed by intracellular *S.* Typhimurium, as has previously been reported in 2D epithelial monocultures (30, 43, 44). However, the relevance of STAT3 activation by *Salmonella* in the context of a complex, multicell-type infection is unclear, partially due to the lack of suitable model systems. Therefore, in the next part of the study, we utilized the 3D tissue model to determine to what degree this major *Salmonella* manipulation strategy impacts host immunity within and beyond the intestinal epithelial compartment.

10.1128/mBio.03348-19.3 FIG S3 Characterization of *Salmonella*-invaded and bystander IEC responses. (A) A Cytoskape network plot showing the top 50 induced RNAs (fold-change ≥ 2, mRNAs and lncRNAs, RNA-seq data) upon *Salmonella* infection in invaded and bystander IECs (Caco-2 cells) and the overlap in gene regulation between the two cell populations. (B) KEGG pathways (*P* values are shown on the *x* axis) of the genes for which the results are shown in panel A induced in invaded and bystander IECs. Pathways marked in red are unique to invaded (GFP-positive) and bystander (GFP-negative) IECs. Download FIG S3, PDF file, 0.2 MB. Copyright © 2020 Schulte et al. 2020 Schulte et al. This content is distributed under the terms of the Creative Commons Attribution 4.0 International license.

10.1128/mBio.03348-19.4 FIG S4 Gene-regulatory networks underlying the IEC-induced genes. (A) Induced network analysis (ConsensusPathDB) with the genes shown in Fig. S3A from invaded (GFP-positive [GFP^+^]) and bystander (GFP-negative [GFP^−^]) IECs. NF-κB and IRF transcription factor-dependent networks were identified in bystander but not in invaded IECs. SOCS3 and chemokine (CXCL2 and -3) networks were induced in invaded IECs. Download FIG S4, PDF file, 0.2 MB. Copyright © 2020 Schulte et al. 2020 Schulte et al. This content is distributed under the terms of the Creative Commons Attribution 4.0 International license.

### T3SS-dependent STAT3 activation creates a locally restricted inflammatory environment.

To study the impact of STAT3 activation by *Salmonella* on the human intestinal environment, we constructed a STAT3-knockout Caco-2 IEC line using CRISPR/Cas9-based genome editing. Western blot analysis confirmed the loss of STAT3 expression in the epithelial lining of barrier models established with these mutant IECs (Fig. 6D, top). Counting of the number of *Salmonella* CFU recovered from infected IECs suggested a reduction in bacterial loads when STAT3 was absent, which, however, did not prove significant at the level of a *P* value of ≤0.05 (Fig. 6D, bottom). qRT-PCR analysis confirmed the expected loss of *SOCS3*, *FGA*, and *FGB* induction in STAT3-deficient *Salmonella*-invaded (GFP-positive) IECs (Fig. 6E). The *Salmonella*-induced downregulation of *GSTA1*, as a control, remained unaltered by STAT3 knockout (Fig. 6E).

**FIG 7:**
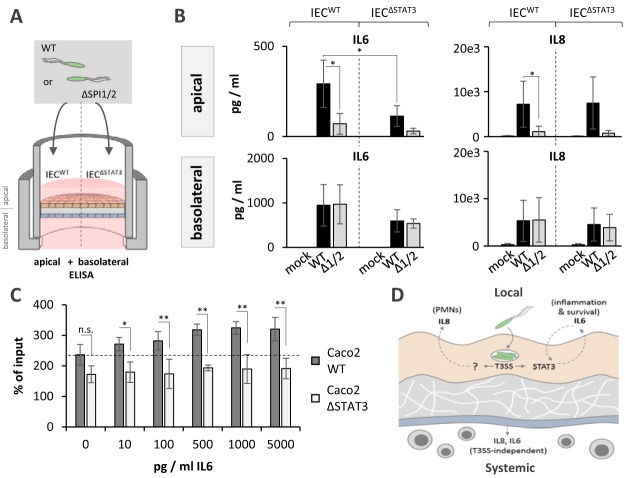
T3SS-dependent effects on the local inflammatory environment. (A) Experimental setup. Intestinal barrier models populated with wild-type (WT) or STAT3-deficient (ΔSTAT3) intestinal epithelial cells were challenged with the *Salmonella* wild-type or ΔSPI1/2 strain. Culture medium from the apical (epithelial) and the basolateral (vascular) compartments was analyzed by ELISA for IL-6 and IL-8 cytokine levels. (B) Results of IL-6 and IL-8 ELISAs, as described in the legend to panel A. Combined results (mean ± SD of secreted cytokine concentrations) from ≥4 independent experiments are shown. *, *P* ≤ 0.05 (two-way analysis of variance). (C) Quantification of *Salmonella* CFU as a percentage of the inoculum recovered from wild-type or STAT3-deficient Caco-2 cells that had been prestimulated with recombinant human IL-6 at the indicated concentrations. Combined results (mean ± SD) from 3 independent experiments are shown. *P* values were determined by a one-way analysis of variance. *, *P* ≤ 0.05; **, *P* ≤ 0.01; n.s., not significant. (D) Schematic representation of manipulation of the host response by *Salmonella*. In the human intestinal system, *S.* Typhimurium infects the epithelium without breaching into the vasculature. Within the epithelial compartment, the pathogen actively shapes the local proinflammatory environment in a T3SS-dependent manner, leading to elevated levels of key cytokines, such as IL-6 and IL-8. While elevated levels of IL-6 in the apical fluid are accomplished through STAT3 targeting, IL-8 levels are modified in a STAT3-independent manner. On the other side of the barrier, the vascular (systemic) cytokine response is independent of the T3SS or of epithelial STAT3. Thus, manipulation of the human immune response by *Salmonella* is locally restricted to the primary site of infection, namely, the epithelial compartment. PMNs, polymorphonuclear leukocytes.

We then measured the inflammatory status on both sides of the barrier in wild-type and STAT3-deficient tissue models by cytokine enzyme-linked immunosorbent assays (ELISAs) for IL-6 and IL-8 (Fig. 7A). IL-6 may amplify STAT3 activation and promote IEC survival (45, 46), whereas IL-8 functions as the major neutrophil attractant (10). To assess the role of *Salmonella* virulence genes in the production of these key proinflammatory cytokines, infections were carried out with either the parental *Salmonella* strain or the ΔSPI1/2 mutant. On the apical side (epithelial compartment), the levels of both cytokines were highly elevated at 24 h after *Salmonella* challenge compared to those after mock treatment (Fig. 7B). Interestingly, IL-6 but not IL-8 levels were markedly reduced in tissue models built with STAT3-knockout IECs compared to the levels in models built with wild-type IECs (Fig. 7B). In contrast, compared to the levels seen upon wild-type *Salmonella* infection, we detected markedly reduced levels of both IL-6 and IL-8 upon infection with the ΔSPI1/2 strain (Fig. 7B). These results suggest that *Salmonella* elevates extracellular IL-6 and IL-8 levels in the epithelial compartment in a T3SS-dependent manner. However, only induction of IL-6 depends upon the *Salmonella*-triggered STAT3 phosphorylation, while IL-8 levels appear to be manipulated through a different mechanism.

Epithelial STAT3 activation seems to promote *Salmonella* infection (43) (Fig. 6D, bottom), which suggests that enforced production of the STAT3 inducer IL-6 serves *Salmonella* to propel the establishment of its infection niche. In line with this assumption, stimulation of wild-type but not STAT3-deficient IECs with recombinant human IL-6 led to elevated *Salmonella* CFU counts (Fig. 7C). This suggests that the *Salmonella* T3SS-mediated, STAT3-dependent elevation of IL-6 levels is part of a self-amplifying, positive-feedback mechanism promoting epithelial infection (Fig. 7D). To determine whether the T3SS-dependent manipulation of cytokine production represents a local *Salmonella* virulence strategy or extends to the vascular immune compartment, IL-6 and IL-8 levels were also measured on the basolateral (vascular) side of intestinal barrier models. Both IL-6 and IL-8 levels were highly elevated in the vascular compartment of apically infected models. Noteworthy was the finding that their induction was largely independent of the presence of both the *STAT3* alleles in epithelial cells and *Salmonella*’s SPI1 and SPI2 virulence gene clusters (Fig. 7B). This reveals a highly localized manipulation of the proinflammatory human host response by *Salmonella* that, different from the situation in infected mice, remains restricted to the intestinal compartment.

## DISCUSSION

Studies of *Salmonella* interaction with human host cells have been largely limited to cell line monocultures or cocultures, such as Caco-2 cells, as an accepted surrogate for the intestinal epithelium (20, 47). These standard culture models, however, cannot fully mimic the complex interaction between *Salmonella* and the different host cell types present on either side of the mucosal/endothelial barrier in the intestine. Advanced, 3D culture methods to model the intestinal lining, e.g., based upon organoid or rotating-wall vessel approaches, have been employed for bacterial infection studies (21); however, they often lack a professional immune cell component or neglect the vascular compartment of the intestinal barrier. Other test systems recapitulate the responses of professional circulating human immune cells while, however, neglecting the epithelial and endothelial cell entities (48).

Organoid cultures, which harbor a physiological mixture of specialized epithelial cell types, have also been employed to study the *S.* Typhimurium interaction with intestinal human cell layers (49, 50). While organoid models represent a promising approach for mimicking bacterium-host interactions at physiologically differentiated gastrointestinal epithelia (51), they cannot be easily modified to contain accessory tissue structures, such as vasculature. Alternatively, modular human organ-on-a-chip models were developed to facilitate, e.g., human disease modeling and drug discovery (52). Despite inclusion of a vascular component and immune cells, these models, however, remain difficult to scale, suffer from a low biomaterial yield for downstream analyses, and use a synthetic instead of a physiological barrier matrix. Alternatively, Transwell-based infection systems populated with apical epithelial and basolateral endothelial cells were proposed to model the intestinal epithelium-blood barrier (53). Such models, however, typically involve artificial collagen-coated membranes, neglecting the complexity and fine structure of the primary intestinal collagen scaffold.

Different from the above-described models, we have introduced here a bioengineered intestinal barrier model in a Transwell-like format composed of a decellularized porcine gut matrix populated with key cellular components, including human IECs, microvascular endothelial cells, and peripheral blood leukocytes. This 3D coculture model allowed us to mimic the interplay of *S.* Typhimurium with major human host cell components during the acute phase of an intestinal infection. To the best of our knowledge, our study is the first to establish human cocultures of epithelium, endothelium, and peripheral blood leukocytes based on a decellularized gut scaffold to reveal bacterial virulence strategies. As we report here, this tissue engineering approach reproduces the epithelium-restricted *S.* Typhimurium infection observed in human patients. While it is possible that the underrepresentation of neutrophils, which may serve *Salmonella* as Trojan horses to transmigrate through the epithelial layer (54), contributes to this observation, microfold (M) cells, through which *Salmonella* may traverse the epithelium, very likely exist in our model, as shown in previous studies and also by us, using electron microscopy (55, 56). It would be an exciting task to study the cellular and molecular basis for epithelial pathogen containment in our model in the future.

We have used our tissue modeling approach to investigate whether and how *Salmonella* virulence strategies discovered in conventional 2D cultures contribute to host manipulation and affect the immune response at the human intestinal barrier. Previously, *Salmonella* has been shown to employ a number of secreted virulence effectors to activate the host STAT3 pathway during its intracellular stage (30, 43, 57, 58). Our previously performed dual RNA-seq analysis with epithelial monocultures allowed us to monitor the expression kinetics of *S.* Typhimurium SPI1 and SPI2 virulence gene clusters during distinct stages of epithelial cell infection and revealed a major role of the *Salmonella* sRNA PinT in timing the transition of virulence programs (30). *S.* Typhimurium has previously been reported to impact the activity of the host JAK/STAT pathway to establish its intracellular infection niche (43). Interestingly, we found that the control of *Salmonella* virulence gene expression by PinT contributes to host JAK/STAT manipulation, likely through altered expression of the important pathway regulator SOCS3 (30). These findings illustrate that dual RNA-seq is capable of identifying bacterial virulence strategies during infection, as well as the counterresponses of the infected host cells.

Our present dual RNA-seq data further corroborate that STAT3 constitutes a central host target of *Salmonella* at the infected intestinal barrier (Fig. 7D), and we reveal how this virulence strategy differentially affects the local and vascular responses. We show that epithelial activation of STAT3 signaling culminates in increased apical IL-6 secretion, which in turn further amplifies STAT3 signaling to promote the survival and proliferation of intestinal epithelial cells (45, 59). *Salmonella*, in turn, benefits from STAT3 activation (57), as increased host cell survival maintains its intracellular replication niche. Besides IL-6, we also noticed the manipulation of IL-8 levels in the apical compartment by *Salmonella* in a T3SS-dependent but STAT3-independent manner. This might be a consequence of the previously reported manipulation of proinflammatory mitogen-activated protein kinase (MAPK) signaling by *Salmonella* (16, 60). Interestingly, the manipulation of cytokine levels remains restricted to the epithelial compartment, as basolateral (vascular) cytokine levels were independent of *Salmonella*’s T3SSs. Induction of the endothelial immune response might in this case involve soluble factors produced by the epithelium or *Salmonella* immune agonists, such as lipopolysaccharide (LPS), traversing across the intestinal barrier.

While our results suggest that *Salmonella* uses its T3SSs to remodel the inflammatory microenvironment at the site of infection, the contribution of the T3SSs to epithelial cell invasion during acute infection remains controversial. Whereas invasion of 2D epithelial cell cultures requires the SPI1 T3SS, in a previous study using epithelial 3D cell cultures induced by a rotating-wall vessel system, an invasion defect was not observed either for a SPI1 T3SS mutant or for a SPI1/2 T3SS double mutant (61). The latter finding contrasts with the observation made here that the SPI1 T3SS is still required for epithelial cell invasion in the 3D intestinal tissue model. These discrepancies might be explained by either *Salmonella* strain-specific differences or disparities in the epithelial cell polarization methods and the extracellular matrix composition between the two studies. Interestingly, the global gene expression profile of intracellular *Salmonella* did not markedly differ from the transcriptomic patterns of this pathogen during growth inside epithelial cells in 2D monocultures (30). This implies that *Salmonella* adaptation of global gene expression in the intracellular environment is independent of the presence or absence of additional host cell types in the infected culture.

We would like to emphasize that the downstream effects of the infection on endothelial and professional immune cells uncovered in our coculture model here were missed in previous infection studies with 2D monocultures. Thus, the spatial restriction of *Salmonella*’s T3SS-dependent epithelial manipulation could be identified only in the present coculture model. Most importantly, this localized manipulation strategy is in line with the epithelium-restricted infection observed in human *in vivo* scenarios but cannot be recapitulated using small-animal models. We are confident that our human intestinal barrier model will be a valuable tool to study the virulence mechanisms of further pathogens for which 2D monocultures are too simplistic to uncover *in vivo*-relevant virulence strategies and for which whole-animal models can only imperfectly reconstitute human disease phenotypes.

The limitations of our model also need to be discussed. Caco-2 cells, which are regarded as a valid IEC model in 3D cocultures (47), do not recapitulate the full complexity of the mucosal epithelium. As an alternative cell source, primary intestinal cells, e.g., cells from biopsy-derived material, can be grown as organoid cultures, while epithelial cell monolayers can be maintained for subsequent implementation into the barrier model (62). Further improvements may be achieved by the use of bioreactor models, applying agitation for a more physiological differentiation and, thus, infection of the epithelium (53, 63). Furthermore, resident mucosal immune cell populations may be integrated (47) to study the role of local antigen-presenting cells in infection and their manipulation by bacterial pathogens. In mice, resident and recruited phagocytes may perturb the epithelial barrier or be hijacked by *Salmonella*, thereby contributing to the systemic dissemination of the pathogen (54). In further iterations of our intestinal model, the contributions of phagocytes to the restriction of *Salmonella* infection to the epithelial compartment in humans could be studied, e.g., by adding or depleting neutrophils or dendritic cells at both sides of the epithelial barrier. Besides improving host cell composition, the apical compartment could be precolonized with bacterial species of the commensal gut microbiota to study competition with and protection against pathogenic invaders. Even if they are further improved in these ways, artificial intestinal barrier models may provide only limited insight into human systemic infection processes.

Humanized mice have been used to study, e.g., systemic infections caused by the human-obligate pathogen *Salmonella* Typhi (64, 65). However, the limited cross-species activity of key immune mediators, such as IL-6 or granulocyte-macrophage colony-stimulating factor (66), and the persisting differences in immune responses by nonimmune cells constitute the major disadvantages of these models. Alternatively, human intestinal tissue models, e.g., models mimicking the blood pulse of the vascular compartment and the peristaltic contractions of the intestine for a more physiological tissue differentiation (62, 67), may be linked to other organ models (e.g., liver or kidney) through bioartificial vasculature to study human infection processes on a more systemic level. Furthermore, recent advances in organ-on-a-chip technology (68–70) may enable the affordable, modular assembly of such communicating human organ systems to mimic systemic infection outcomes. While these potential improvements may bring bioartificial human infection models closer to actual physiological conditions, they also add additional complexity and are difficult to control by nonexpert users. We thus regard our present model to be a reasonable compromise between complexity and broad applicability.

Follow-up studies may combine our tissue modeling approach with other emerging technologies to advance the understanding of infectious diseases at the molecular level. For example, here we have used flow sorting and dual RNA-seq to study the reciprocal adaptation of host and pathogen gene expression at the intestinal barrier. Further resolution may be obtained by combining our model with single-cell RNA-seq (71) to chart the communication of individual cells at both sides of the infected intestinal barrier, without limitation to defined cell populations. Our model may also constitute an attractive platform for subsequent human gene loss-of-function studies, using the CRISPR/Cas9 technology, to address potential therapeutic interventions at the intestinal barrier. Altogether, we are confident that the present approach will advance research on human infectious diseases and antimicrobial strategies beyond *Salmonella* infections.

## MATERIALS AND METHODS

### Tissue model setup.

Colorectal Caco-2 cells were cultured in cell culture flasks using minimum essential medium (MEM) supplemented with 20% fetal calf serum (FCS), 1% sodium pyruvate, and 1% nonessential amino acids, until seeding onto the collagen scaffold. Microvascular endothelial cells from foreskin were obtained via a previously published protocol (72) and expanded in VascuLife vascular endothelial growth factor (VEGF)-Mv medium (Lifeline Cell Technology) until seeding onto the collagen scaffold (72). Primary human blood leukocytes were isolated from fresh buffy coats by Lymphoprep (Axis Shield) gradient centrifugation according to the manufacturer’s instructions. Upon two washes with phosphate-buffered saline (PBS), the leukocytes were resuspended in 1:1 VascuLife (Lifeline Cell Technology)–X-Vivo-15 (Lonza) medium. Tissue models were based on modified biological vascularized scaffolds (BioVaSc) (62) fixed in custom-made cell crowns with a surface area of 1.1 cm^2^, separating the apical compartment from the basolateral compartment. Caco-2 cells were seeded on the mucosal side of the biological scaffold with a preserved crypt and villus structure at a density of 0.3 × 10^6^ cells per cell crown and cultured for 21 days under static culture conditions. Cell culture medium (as described above) was exchanged three times a week. On day 17, the barrier integrity of the models was determined by analysis of fluorescein isothiocyanate (FITC)-dextran permeation (4 kDa; Sigma-Aldrich). Models with a relative permeability of >1.5% were discarded. On day 18, 0.4 × 10^6^ endothelial cells were seeded on the basolateral side of the scaffold in a medium volume of 20 μl. After an incubation period of 1 to 2 h to allow the endothelial cells to adhere, Caco-2 cell medium (see above) was added to the apical compartment and VascuLife VEGF-Mv medium (Lifeline Cell Technology) was added to the basolateral compartment. Leukocytes (10^6^) were supplemented into the basolateral compartment (6-well format, 2-ml total volume of 1:1 VascuLife–X-Vivo-15 medium) on the day of the infection experiment.

### *Salmonella* infection assays.

Cells of GFP-expressing *S.* Typhimurium strain SL1344 (29) or an SPI1/SPI2-deficient mutant thereof (73) were grown to an optical density at 600 nm of 2.0 in LB medium at 37°C with shaking at 180 rpm. Upon one wash in Caco-2 cell medium, bacteria were supplemented into the apical compartment of the barrier model at a multiplicity of infection (MOI) of 10 (i.e., 10 bacteria/Caco-2 cell). Extracellular bacterial replication was prevented by addition of gentamicin (final concentration, 20 μg/ml) into the apical compartment at 1 h postinfection. At the time points after infection indicated above (see text and figures in Results), epithelial and endothelial cells were collected directly from the scaffold by trypsin-Accutase cell detachment solution treatment upon 2 washes with PBS. Leukocytes were collected directly from the basolateral compartment and further purified by cell sorting (see below). For CFU assays, Caco-2 cells were lysed with PBS containing 0.01% Triton X-100. The lysates were serially diluted in PBS and plated onto LB agar, followed by overnight incubation at 37°C. Control samples were mock treated (mock treatment was same treatment used for the infected samples but with the addition of sterile medium instead of the bacterial suspension).

### CRISPR/Cas9-based genome editing.

A synthetic DNA segment (Metabion; see Table S2 in the supplemental material) was cloned into the BbsI site of the pX458 CRISPR vector (from the F. Zhang lab [74] through Addgene) for expression of a guide RNA targeting the *STAT3* coding sequence. Caco-2 cells were transfected with 1 μg of plasmid DNA using the Lipofectamine 2000 reagent (Thermo Fisher) according to the manufacturer’s instructions. At 24 h after transfection, single transfected (GFP-positive) cells were spotted into 96-well plates that had been prefilled with complete medium containing 100 μg/ml of the Normocin antibiotic mixture (Invivogen), using a FACSAria III cell sorter (BD) with a 100-μm nozzle size. During clonal expansion in the wells of the 96-well plate, fresh medium was added every 5 days. Knockout success was evaluated by PCR amplification of the *STAT3* coding sequence from genomic DNA and confirmed by Sanger sequencing (Seqlab GmbH, Göttingen, Germany).

10.1128/mBio.03348-19.6 TABLE S2 Oligonucleotides used in the present study. Download Table S2, PDF file, 0.1 MB. Copyright © 2020 Schulte et al. 2020 Schulte et al. This content is distributed under the terms of the Creative Commons Attribution 4.0 International license.

### Quantitative real-time PCR.

qRT-PCR analyses were carried out using a Power SYBR green RNA-to-Ct 1-step kit (Thermo Fisher) according to the manufacturer’s instructions and a QuantStudio3 real-time PCR machine (Applied Biosystems). RNA was extracted using the TRIzol reagent (Thermo Fisher) method. To remove genomic DNA, the extracted nucleic acids were incubated with DNase I (Thermo Fisher) and an RNase inhibitor (Promega) for 30 min at 37°C and subsequently extracted with phenol-chloroform-isoamyl alcohol (Sigma-Aldrich), followed by precipitation with 30:1 ethanol–5 M sodium acetate. The qRT-PCR primers are listed in Table S2. Fold changes based on threshold cycle (*C_T_*) values were calculated using the 2^−ΔΔ^*^CT^* method (75), and human U6 snRNA was used as an internal reference.

### Immunostaining and flow cytometry.

Cells collected from the barrier model were analyzed using a FACSCalibur or a FACSAria III device (BD). To purify monocytes and NK cells from the collected leukocytes, the cells were stained with anti-CD14-FITC (catalog number 11-0149-42; Thermo Fisher) and anti-CD56-allophycocyanin (catalog number 17-0567-41; Thermo Fisher) antibodies in PBS, 0.1% FCS and sorted using a FACSAria III device (100-μm nozzle, single-cell purity setting). FCS3.0 files were analyzed using Flowing software (http://flowingsoftware.btk.fi/).

### Dual RNA-seq and computational analyses.

For RNA-seq analysis, cellular RNA was extracted using a *mir*Vana RNA isolation kit (Thermo Fisher) according to the total RNA isolation protocol supplied with the kit. rRNA was depleted using a Ribo-Zero gold (epidemiology) kit (Illumina). Libraries were generated and sequenced on a NextSeq 500 platform at Vertis Biotech (Freising, Germany) as previously described (30). Demultiplexed reads were mapped to the GRCh38 human reference annotation using the CLC Genomics Workbench (Qiagen) with standard settings (mismatch cost = 2, insertion cost = 3, deletion cost = 3, length fraction = 0.8, similarity fraction = 0.8). The data tables obtained were filtered for genes with a number of reads per kilobase per million (RPKM) value of ≥0.5 in both sequenced replicates under at least one experimental condition. Genes exhibiting fold changes in expression of ≥2 or ≤0.5 (calculated based on RPKMs) in both replicates were considered differentially expressed. Hierarchical clustering was performed using the Cluster program (Michael Eisen lab) with the correlation (uncentered) similarity metric and the centroid linkage clustering method. Heat maps were generated using the Java TreeView program (76). PCA analysis was done in R software using the script prcomp (stats) and the rgl package. Network plots were generated with Cytoscape software (version 3.7.1). KEGG pathway analysis and induced network analysis were performed using the ConsensusPathDB molecular functional interaction database (77).

Bacterial bioinformatics analyses (Fig. 5B and C) were performed as follows. Samples including genes with an RPKM of >1 in at least one sample and a coefficient of variation of >0.5 were subjected to a 3-dimensional principal-component analysis (with the R software scatterplot3d package, version 0.3-41), using log_2_(RPKM) values as the input. Unsupervised complete linkage clustering (with the R software heatmap.2 function from the gplots package, version 3.0.1.1) was performed on rows and columns using the Euclidian distance as a similarity metric and log_2_(RPKM) values as the input. *Salmonella* GO term enrichment analysis (Fig. 5C) was performed using the ShinyGO tool (version 0.60; http://bioinformatics.sdstate.edu/go/) for the GO term biological process with an FDR cutoff of 0.05.

### Western blot analysis.

For Western blot analysis, samples were collected in radioimmunoprecipitation assay buffer supplemented with Laemmli buffer and boiled for 5 min. Proteins were separated on 10% polyacrylamide-SDS gels and transferred onto nitrocellulose membranes (catalog number 10600015; Amersham) by semidry blotting. Proteins were detected using anti-STAT3 (catalog number 9139; Cell Signaling), anti-phospho-STAT3 (catalog number 9134; Cell Signaling), and anti-actin (catalog number sc-1616; Santa Cruz) primary antibodies, horseradish peroxidase-linked secondary antibodies, and an enhanced chemiluminescence (ECL) reagent (catalog number RPN2232; Amersham). Images were obtained using an Intas Advanced ECL imager system.

### Histology.

Tissue samples were fixed with 4% paraformaldehyde for 1 h at 4°C. Samples were embedded in paraffin and sectioned to a thickness of 5 μm with a microtome (model SM2010 R; Leica). Tissue slices were first deparaffinized using the Roticlear clearing agent (Carl Roth) and rehydrated in a graded series of ethanol according to standard protocols. Characterization of the tissue samples was done by immunofluorescence staining. For antigen retrieval, tissue slices were heat pretreated at 100°C for 20 min in pH 6 citrate buffer (Carl Roth). After blocking unspecific binding by PBS with 0.3% Triton X-100 (Sigma-Aldrich), 5% bovine serum albumin (BSA; PanReac AppliChem), and 5% donkey serum (Biozol) for 30 min, the slices were incubated with primary antibodies at 4°C overnight. The following primary antibodies were used at a 1:100 dilution: pan-cytokeratin (pCK; specific for epithelial cells; Dako), CD31 (endothelial cell specific; Abcam), and LPS (for the detection of *Salmonella*; Abcam). After washing, anti-mouse/anti-rabbit immunoglobulin-Alexa Fluor 555 and -Alexa Fluor 647 secondary antibodies were added at a dilution of 1:400 in antibody dilution solution, and the mixture was incubated for 1 h at room temperature. Samples were mounted using Mowiol mounting medium with DAPI (4′,6-diamidino-2-phenylindole; Sigma-Aldrich) for nuclear staining. Imaging was achieved using an inverted fluorescence microscope (Keyence BZ-9000).

### ELISA.

Enzyme-linked immunosorbent assays (ELISAs) were performed using human IL-6 (catalog number 88-7066-86) and IL-8 (catalog number 88-8086-86) Ready-Set-Go ELISA kits (Thermo Fisher) according to the manufacturer’s instructions. The cell culture supernatants were centrifuged for 1 min at maximal speed to pellet the cell debris, and samples were used at a 1:10 (IL-6) or 1:150 (IL-8) dilution. The samples were analyzed using a Tecan Sunrise plate reader, and absolute quantification was achieved using the cytokine standards supplied with the ELISA kit.

### Data availability.

RNA-seq data have been uploaded to the NCBI GEO repository (GEO accession number GSE136717).
