# Supplementary figures and images for: An Advanced Human Intestinal Coculture Model Reveals Compartmentalized Host and Pathogen Strategies during Salmonella Infection
Source: mBio. 2020 Feb 18;11(1):e03348-19. doi: 10.1128/mBio.03348-19 (PMC7029144; doi:10.1128/mBio.03348-19)

Fig. S1

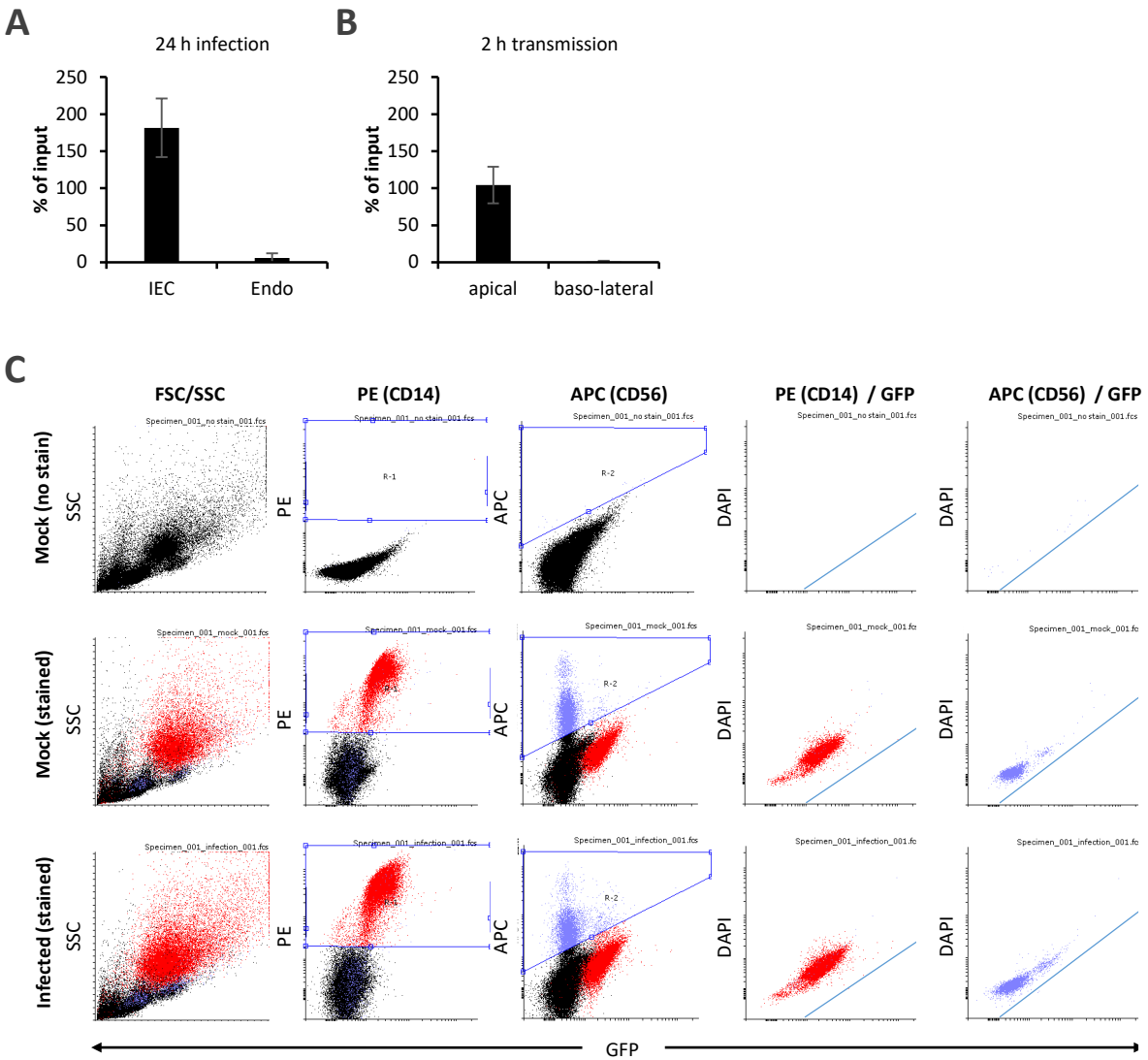

Supplement: FIG S1 [file mBio.03348-19-sf001.pdf]

Fig. S2

**A**

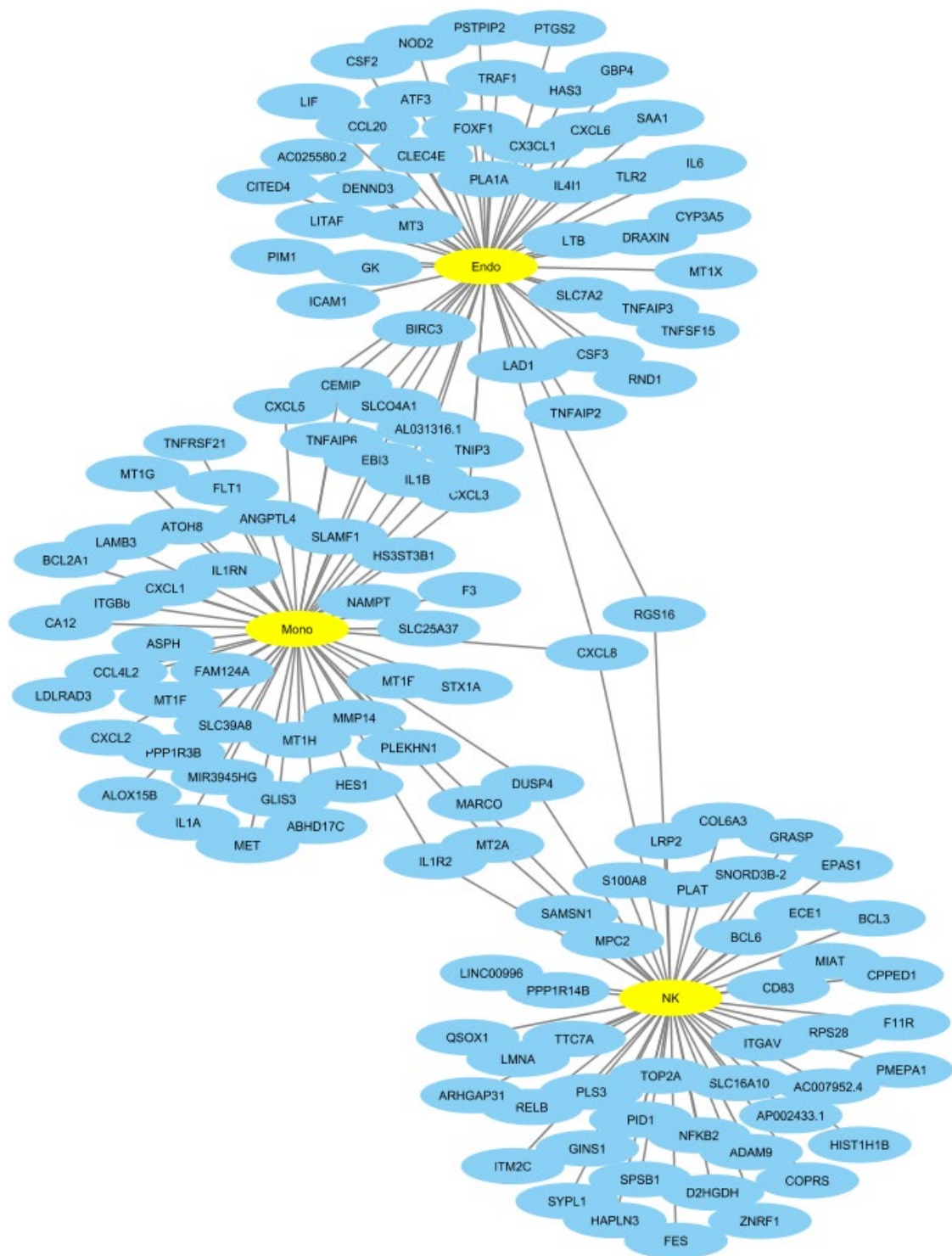

Supplement: FIG S2 [file mBio.03348-19-sf002.pdf]

Fig. S3

A

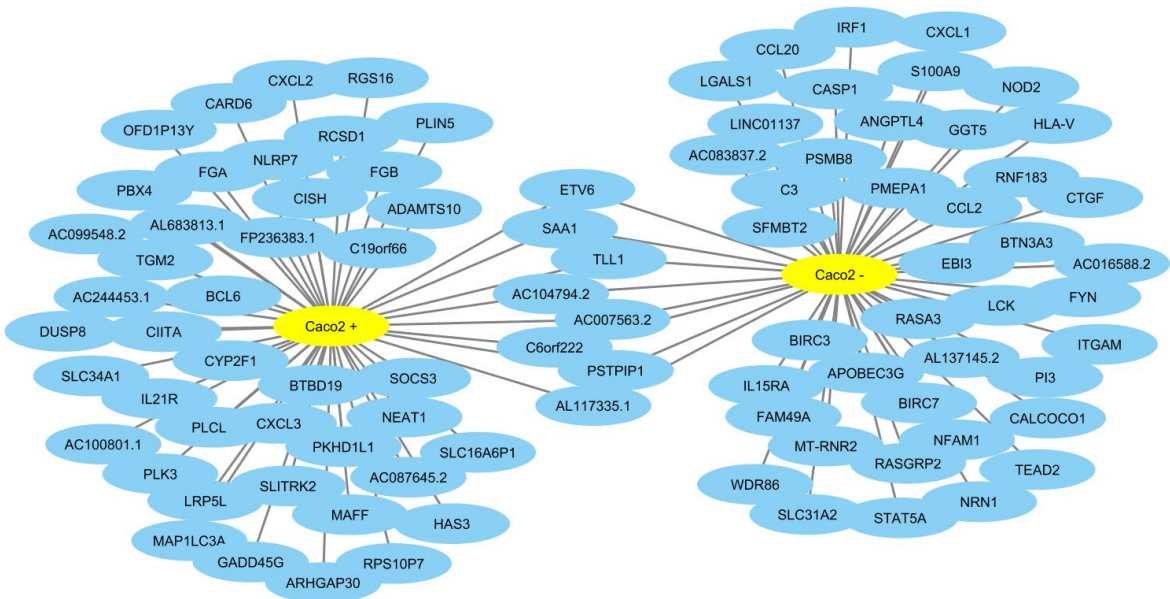

B

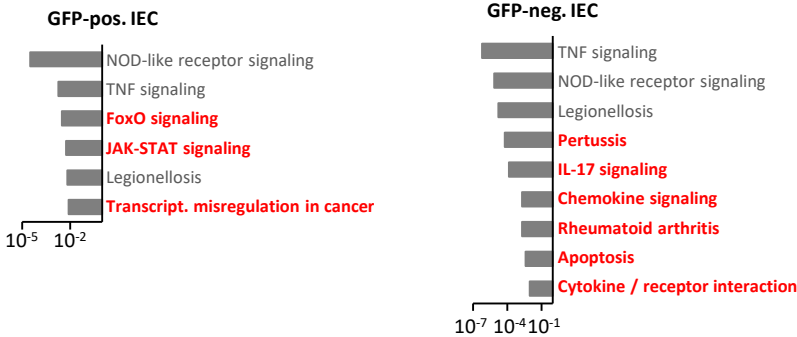

Supplement: FIG S3 [file mBio.03348-19-sf003.pdf]

Fig. S4

A

GFP+ (Salmonella invaded IEC) induced network:

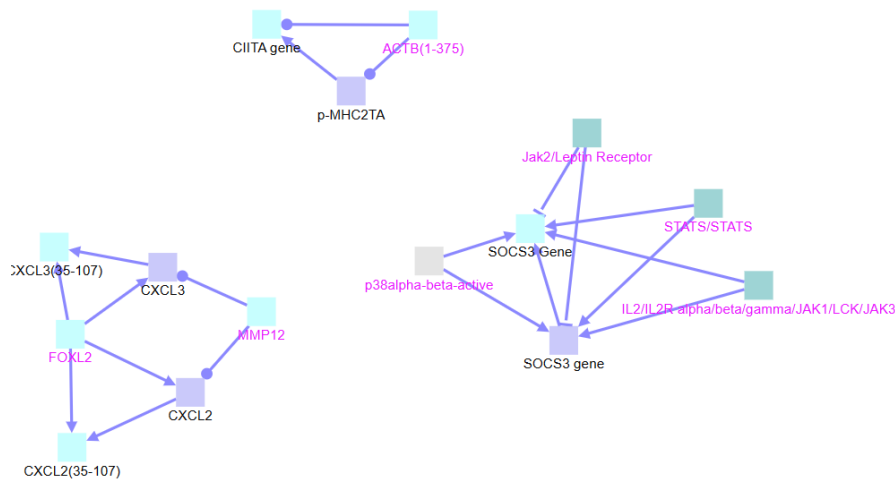

GFP- (bystander IEC) induced network :

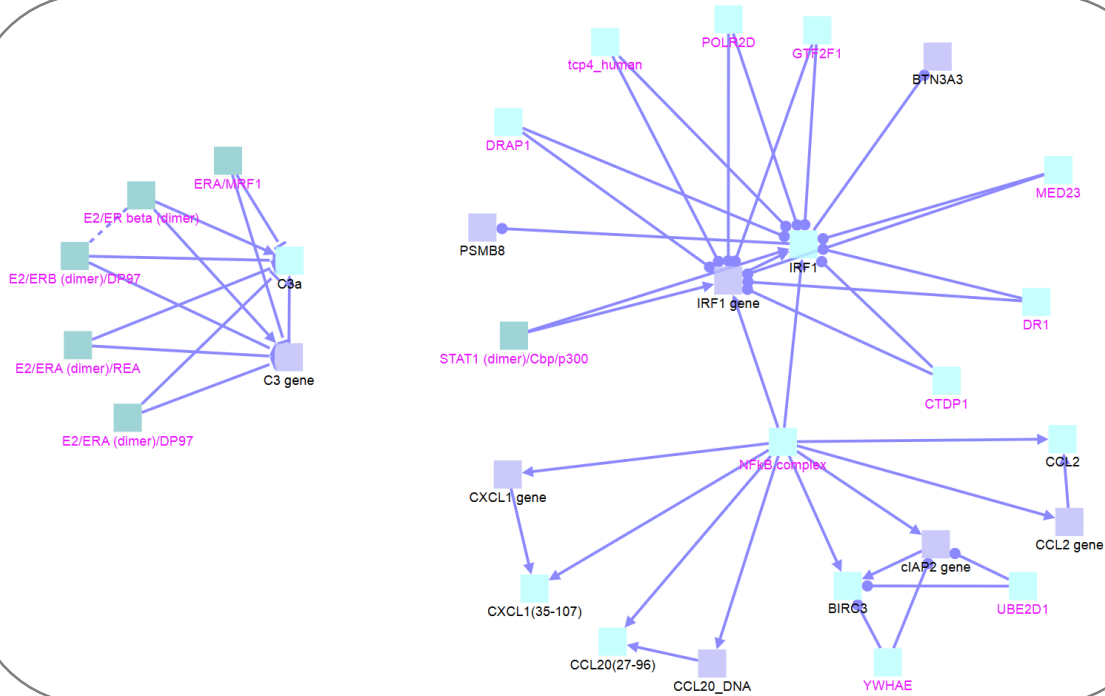

Supplement: FIG S4 [file mBio.03348-19-sf004.pdf]
