# Supplementary material for: An Advanced Human Intestinal Coculture Model Reveals Compartmentalized Host and Pathogen Strategies during Salmonella Infection
Source: mBio. 2020 Feb 18;11(1):e03348-19. doi: 10.1128/mBio.03348-19 (PMC7029144; doi:10.1128/mBio.03348-19)
Supplement: TABLE S1 [file mBio.03348-19-st001.pdf]

Table S1

Table S1: Differentially regulated *Salmonella* sRNAs in invaded IECs, compared to inoculum

Gene ID	fold-change (replicate 1)	fold-change (replicate 2)
AmgR	1403.964	1117.446
PinT	112.5617	257.1516
STnc510	54.94968	51.03295
StyR-3	36.3856	14.78196
RyhB	38.09845	11.77902
RUF_341c.10	12.64595	35.99503
IsrE	22.96405	13.97454
RybA	17.61746	15.38611
IsrC	12.48856	16.24391
STnc800	3.26151	20.39718
STnc3080	16.22009	4.921354
STnc650	4.592809	12.94915
SLnc0002	9.862763	6.091466
MgrR	4.607147	8.749353
StyR-281	5.594877	7.673358
IsrG	5.827996	5.27927
STnc3150	6.203996	4.167845
RyfA	3.854186	6.474577
sRNA10	6.519454	2.639635
STnc1990	4.226899	4.399392
rpsL-leader	3.628753	4.799337
STnc780	2.271542	3.885757
STnc500	2.530577	2.140245
STnc610	0.384648	0.417517
RprA	0.420738	0.366805
CsrB	0.33927	0.371377
STnc2130	0.352242	0.356707
StyR-44b	0.498248	0.072319
RUF_257c	0.416466	0.130891
IsrK	0.363561	0.112805
RyfD	0.336651	0.118902
CsrC	0.26415	0.153775
zipA-leader	0.246569	0.163276
StyR-29	0.249663	0.156539
STnc3680	0.167968	0.229533
StyR-55	0.212689	0.166714
STnc1460	0.196695	0.150668
tpk3	0.149824	0.152901
DapZ	0.224572	0.062292
SLnc0014	0.068083	0.194277
GcvB	0.101034	0.141371
InvR	0.05806	0.032009
