# Supplementary material for: An Advanced Human Intestinal Coculture Model Reveals Compartmentalized Host and Pathogen Strategies during Salmonella Infection
Source: mBio. 2020 Feb 18;11(1):e03348-19. doi: 10.1128/mBio.03348-19 (PMC7029144; doi:10.1128/mBio.03348-19)
Supplement: TABLE S2 [file mBio.03348-19-st002.pdf]

Table S2

Table S2: Oligonucleotides used in the present study

Target	Oligo name	Oligo sequence
**hSTAT3 (pX458 inserts)**	OBS-1230	Sense: CACCGAATGGAGATTGCCCGGATTG
	OBS-1231	Antisense: AAACCAATCCGGGCAATCTCCATTC
**hU6 (qRT-PCR)**	OBS-0712	Fwd: GCTTCGGCAGCACATATACTAAAAT
	OBS-0713	Rev: ATATGGAACGCTTCACGAATTTG
**hIL6 (qRT-PCR)**	OBS-0015	Fwd: AATTCGGTACATCCTCGACGG
	OBS-0016	Rev: TTGGAAGGTTCAGGTTGTTTTCT
**hCSF2 (qRT-PCR)**	OBS-1576	Fwd: GAGCCGACCTGCCTACAGA
	OBS-1577	Rev: TCTGGGTTGCACAGGAAGTT
**hLUCAT1 (qRT-PCR)**	OBS-849	Fwd: ACCATGTGTCAAGCTCGGATTG
	OBS-850	Rev: TTGTGGTCTCTGGTGCCAAG
**hMSC-AS1 (qRT-PCR)**	OBS-2243	Fwd: TGCCACGTCCAAGGTTCAG
	OBS-2244	Rev: AGGCTTCTTCCATGGCAGG
**hCXCL5 (qRT-PCR)**	OBS-2245	Fwd: TCCTTCGAGCTCCTTGTGC
	OBS-2246	Rev: ATGAACTCCTTGCGTGGTCTG
**hCA12 (qRT-PCR)**	OBS-2247	Fwd: ATAGACCTGCACAGTGACATCC
	OBS-2248	Rev: TGGCACTGTAGCGAGACTG
**hMIR3945HG (qRT-PCR)**	OBS-2249	Fwd: TCACCGCTGTGTCTAGGTG
	OBS-2250	Rev: AGTTCTCCAGTCCTCAGCAC
**hPID1 (qRT-PCR)**	OBS-2309	Fwd: AAGCCAGTCATTGAGCTCTGG
	OBS-2310	Rev: TTGTGGTCGAGATGATGGAGC
**hCXCL8 (qRT-PCR)**	OBS-0017	Fwd: ACTGAGAGTGATTGAGAGTGGAC
	OBS-0018	Rev: AACCCTCTGCACCCAGTTTTC
**hMIAT (qRT-PCR)**	OBS-2311	Fwd: AACTGCTATTAGAAGGAGGCTGG
	OBS-2312	Rev: TAACTCTAAGGCCAAGGAGCC
**AC007952.4 (qRT-PCR)**	OBS-2313	Fwd: TTCTCCTGAGCGTGAAGCC
	OBS-2314	Rev: AACCACTCAGACCGCGTTC
**hFGA (qRT-PCR)**	OBS-1824	AGCCGATCATGAAGGAACAC
	OBS-1825	ACTGGTCTCTTGATCGCAAT
**hFGB (qRT-PCR)**	OBS-1826	TGATTCAGAACCGTCAAGAC
	OBS-1827	TCCCATCCTGGTAAGCTGGC
**hSOCS3 (qRT-PCR)**	OBS-1830	CCGTCTCGCCGCCGCGTCGC
	OBS-1831	ATGGCGCACGGAGCCAGCGT
**hGSTA1 (qRT-PCR)**	OBS-1836	AACGCAGTCATGGAGGCCAA
	OBS-1837	AACTAAGTCAGCGAATAGGA
**hU6 (Northern blot)**	JVO-7672	ATATGGAACGCTTCACGAATTTG
**S.tm_5S (Northern blot)**	JVO-322	CTACGGCGTTTCACTTCTGAGTTC
**S.tm_DapZ (Northern blot)**	JVO-8994	CAATTAATCTTGATTACCACAACCA
**S.tm_InvR (Northern blot)**	JVO-222	GATAAATGCAACGTAAGAGACAAATG
**S.tm_IsrE (Northern blot)**	JVO-9807	AGGCGGGCAAATAATACTG
**S.tm_PinT (Northern blot)**	JVO-2408	TATGAGGAGGACAATTACCG
